# Functions of the ventromedial prefrontal cortex in emotion regulation under stress

**DOI:** 10.1038/s41598-021-97751-0

**Published:** 2021-09-14

**Authors:** Yukihiro Suzuki, Saori C. Tanaka

**Affiliations:** 1 Faculty of Information Science, Nara Institute of Science and Technology, Nara, Japan; 2 Brain Information Communication Research Laboratory Group, Advanced Telecommunications Research Institutes International, Kyoto, Japan

**Keywords:** Prefrontal cortex, Stress and resilience

## Abstract

Recent neuroimaging studies suggest that the ventromedial prefrontal cortex (vmPFC) contributes to regulation of emotion. However, the adaptive response of the vmPFC under acute stress is not understood. We used fMRI to analyse brain activity of people viewing and rating the emotional strength of emotional images after acute social stress. Here, we show that the vmPFC is strongly activated by highly emotional images, indicating its involvement in emotional regulation, and that the midbrain is activated as a main effect of stress during the emotional response. vmPFC activation also exhibits individual differences in behavioural scores reflecting individual reactions to stress. Moreover, functional connectivity between the vmPFC and midbrain under stress reflects stress-induced emotion regulation. Those results suggest that the functions of the network including the vmPFC in emotion regulation is affected by stress depending on the individuals' level of reaction to the stress.

## Introduction

Stress is inevitable. When stressed, we often experience undesired negative emotions, such as fear, anger, or sadness. Successful regulation of emotions is essential to cope properly with stress; however, the effect of stress on emotion regulation is unclear.

Emotion regulation has been defined as an attempt to monitor and modulate emotional experience^1^. Emotion regulation is also involved in cognitive functions, such as decision making, and is thought to reduce risk behaviour^2^ and to influence behaviour in the social decision paradigm^3^. Impaired emotion regulation is exhibited by patients with mental disorders, such as depression and autism spectrum disorder, and with adjustment disorders characterised as excessive responses to stress^4,5^.

At the neural level, emotion regulation is associated with anterior parts of the default mode network, the medial prefrontal cortex (mPFC)^6,7^, suggesting that default mode network dysfunction can lead to neuropsychiatric disorders and may impair emotion regulation^8^. In particular, the ventromedial prefrontal cortex (vmPFC), which is part of the mPFC, functions in emotion regulation by encoding emotional stimuli and by regulating anxiety and fear extinction^9–12^. Detailed mechanisms of vmPFC in emotion regulation have been investigated in both rodents and humans^13^. In humans, recent studies using magnetic resonance spectroscopy (MRS) suggest neurochemical functions of the vmPFC in emotion processing^14–17^. In addition, the vmPFC is also involved in stress^18,19^. A recent study suggested a dynamic change in the vmPFC during stress^20^: vmPFC activation initially decreased with exposure to stressful images, but then increased with stress coping. Taken together, the vmPFC appears to be involved in adaptive responses, including emotion regulation, during stress, although detailed mechanisms are largely unknown.

We hypothesised that stress affects vmPFC activation, thereby affecting emotion regulation involving the vmPFC, and that the level of effect is influenced by the individuals' level of reaction to the stress. To examine this hypothesis, we conducted a functional magnetic resonance imaging (fMRI) experiment in which participants viewed emotional images and rated the emotional impact of the images after the induction of acute social stress. Given its association with the receipt of emotional stimuli and their subsequent evaluation, the vmPFC can be involved in stimulus-driven emotional regulation, whereas the limbic system can be activated as an emotional response^10,21,22^. However, some studies suggested that brain regions related to emotional stimuli and response can be mixed^23,24^. Thus, to explore detailed functions of the vmPFC, we focused on activity and connectivity of the vmPFC while participants viewed images and rated their emotional strength. We defined participant levels of response to stress according to the State-Trait Anxiety Inventory, which is frequently used in stress studies. We found that the vmPFC exhibits higher activation for images with high valence and that the brain region associated in stress is involved in the emotion regulation function of the vmPFC.

## Results

**Figure 1:**
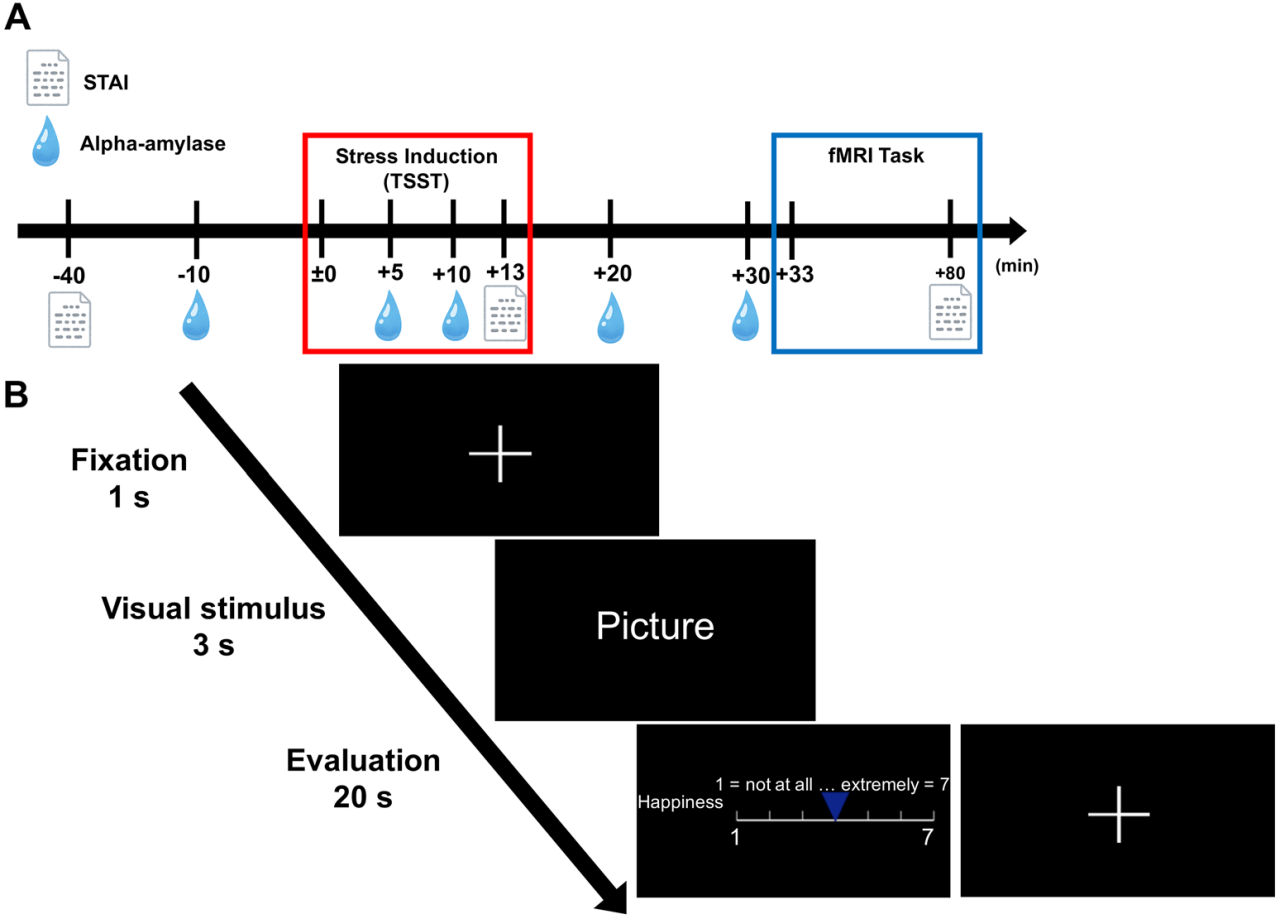
Experimental design and fMRI task. (**A**) Experimental procedure. Both stress and control conditions had the same schedule, but the content of the TSST differed between the two conditions. Onset (± 0 min) was set as the start of the stress test. (**B**) The emotion evaluation task in MRI. Participants underwent an emotion evaluation on a 7-point scale for photographic images displayed on the screen. Evaluations comprised a preference and six basic emotions (happiness, sadness, fear, anger, disgust and surprise). There were four sessions, and 19 images were evaluated in each session.

Twenty-one young males (mean ± SD, 23.10 ± 1.81 years) with no psychiatric disorders, claustrophobia, or neuroendocrine disorders were recruited for two experimental days. Since female stress responses are altered by menstrual cycles^25^ (Kajantie et al., 2006), we enrolled only male participants in this study, as in previous studies^26^ (see the “Discussion” for this limitation). Each day comprised a stress induction test and an fMRI task (Fig. 1A). On the first day (stress condition), all participants completed the Trier Social Stress Test (TSST)^27^, which induces acute social stress and comprises three parts: preparation of an interview about future work (5 min), an interview about future work (5 min), and a difficult arithmetic task. After the TSST, participants conducted an emotion task in the MRI scanner. Participants looked at emotional photographic images and evaluated the strengths of their six basic emotions (happiness, sadness, fear, anger, disgust, surprise) and preferences for the images (Fig. 1B, see “Methods”). On the second day (control condition), participants underwent the same procedure as on the first day, except for the stress induction test. Consistent with a previous study^28^, participants performed free speech and easy arithmetic tasks. We also evaluated the physiological and psychological responses to stress by measuring the State-Trait Anxiety Inventory-JYZ (STAI-JYZ; a self-reported assessment)^29^ and salivary alpha-amylase levels, respectively, multiple times before and after stress induction.

### Physiological and psychological responses to stress

**Figure 2:**
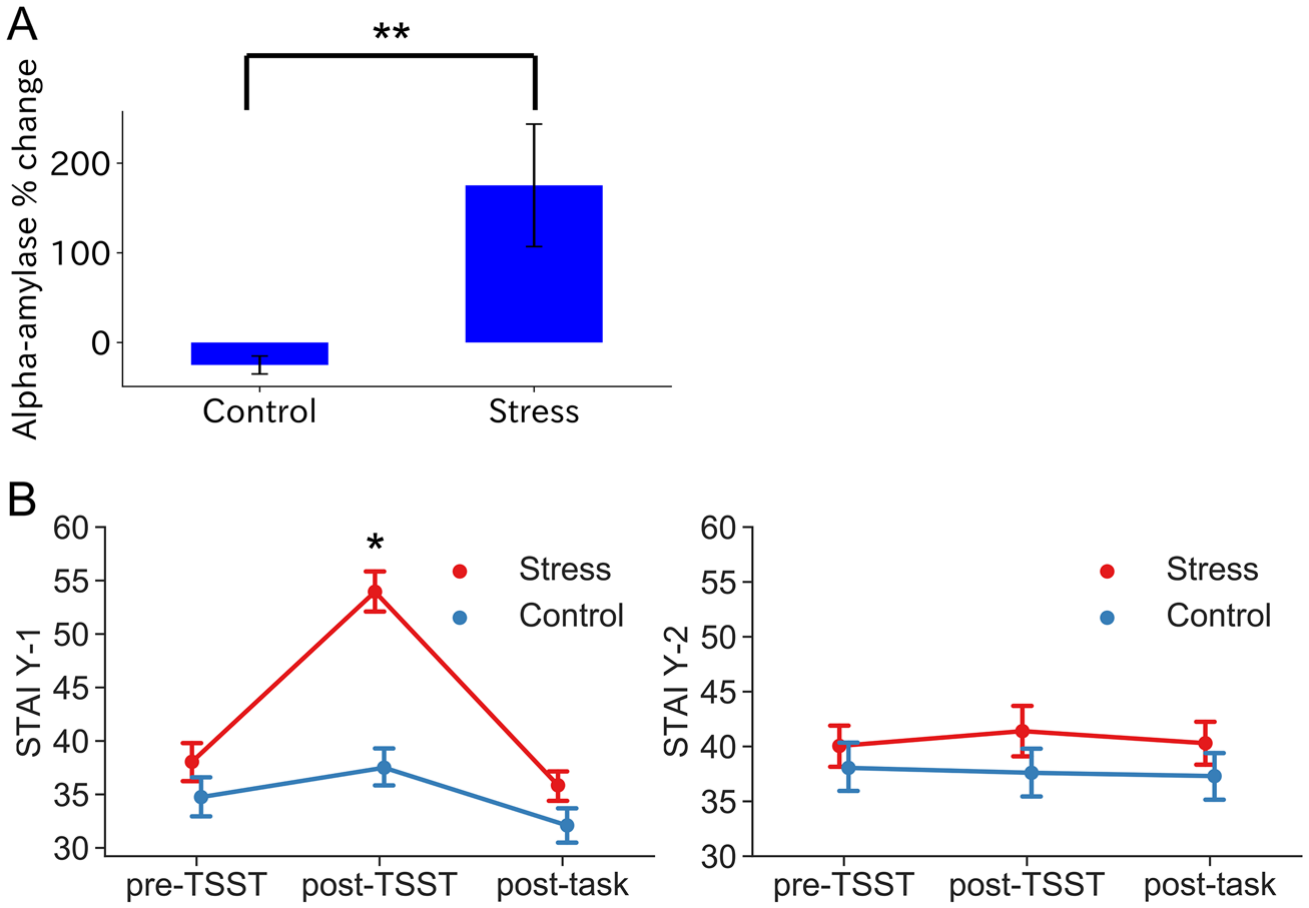
Salivary alpha-amylase levels in STAI-Y1 (state anxiety) and STAI-Y2 (trait anxiety). (**A**) Acute social stress significantly increased the alpha-amylase level (*P* < 0.01 on paired *t*-test). The alpha-amylase level represents the percent change between − 10 min (before the TSST) and + 10 min (after the TSST speech). (**B**) The STAI Y-1 showed a significant condition (stress, control) × time (− 40 min, after the TSST, + 80 min) interaction. (F_(2,114)_ = 8.68, *P* < 0.001 in two-way repeated measures ANOVA). However, the STAI Y-2 showed no significant difference (*P* > 0.05). Error bars indicate s.e.m.

To test whether our experimental procedure could provoke stress in participants, we measured salivary alpha-amylase several times during the experiment, because salivary alpha-amylase levels are increased by acute stress^30^. We set the onset (± 0 min) as the start of the TSST and measured saliva at − 10 min, + 5 min, + 10 min, + 20 min and + 30 min (Fig. 1A). We calculated the percentage change in salivary alpha-amylase between − 10 min (before the TSST) and + 10 min (after the final speech during the TSST) under both stress and control conditions. Under stress, salivary alpha-amylase was significantly increased compared with the control condition (*t*(20) = 3.09, *P* < 0.01 on paired *t*-test; Fig. 2A). Thus, stress was thus successfully induced under the test conditions.

To assess the effect of stress on psychological aspects, we measured the STAI-JYZ at − 40 min (before the TSST), + 13 min (after the TSST) and + 80 min (after the fMRI task) (Fig. 1A and “Methods”). STAI Y-1 (state anxiety) showed significant interactions between condition (stress, control) × time (− 40 min, + 13 min, + 80 min) (F_(2,114)_ = 8.68, *P* < 0.001 on two-way repeated measures ANOVA; Fig. 2B), but no significant interaction or main effects were found in STAI-Y2 (trait anxiety). In addition, Tukey's HSD multiple comparison test revealed a difference in state anxiety immediately after the TSST between stress and control conditions. This indicated that state anxiety was induced only by acute stress, but did not persist. Therefore, a brief stress state was provoked by the TSST (acute social stress).

### Subjective evaluations in the fMRI task

**Figure 3:**
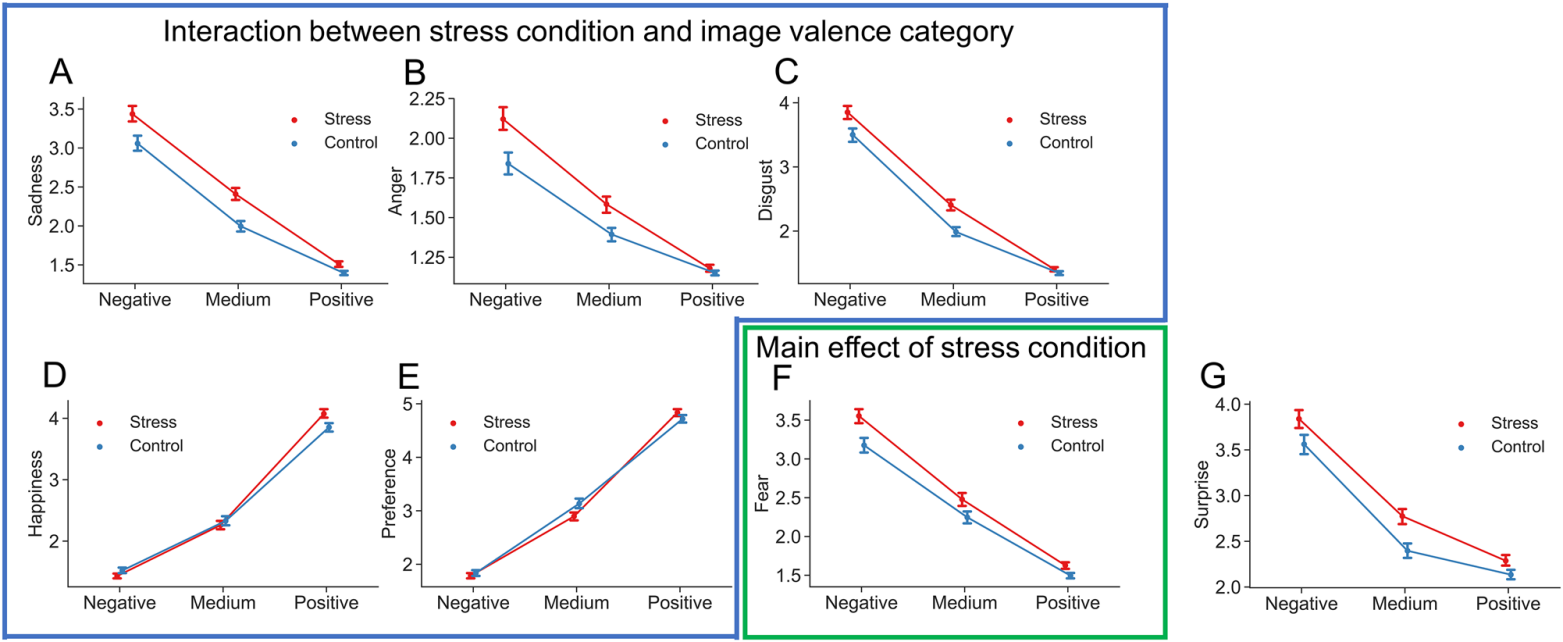
Plot of evaluation scores for valence assigned to images (negative, medium, positive) in stress (red) and control (blue) conditions. Sadness, anger, disgust, preference, and happiness showed significant valence × condition interactions (F_(2,40)_ = 9.06, *P* < 0.001; F_(2,40)_ = 2.82, *P* < 0.05; F_(2,40)_ = 4.99, *P* < 0.01; F_(2,40)_ = 3.81, *P* < 0.05; F_(2,40)_ = 6.24, *P* < 0.01, respectively, in two-way repeated measures ANOVA). Evaluations of fear and surprise did not reveal a valence × condition interaction, but fear showed a main effect of condition (F_(2,20)_ = 7.89, *P* = 0.011). Error bars indicate the s.e.m.

We conducted two-way ANOVA of all participant evaluation scores of strengths of their six basic emotions (happiness, sadness, fear, anger, disgust, surprise) and preferences with factors of valence assigned to the images (negative, medium, positive) and conditions (stress, control). Sadness, anger, disgust, preference and happiness showed significant valence × condition interactions (F_(2,40)_ = 9.06, *P* < 0.001; F_(2,40)_ = 2.82, *P* < 0.05; F_(2,40)_ = 4.99, *P* < 0.01; F_(2,40)_ = 3.81, *P* < 0.05; F_(2,40)_ = 6.24, *P* < 0.01, respectively, in two-way repeated measures ANOVA; Fig. 3). Fear and surprise did not show a significant valence × condition interaction, but fear showed a main effect of condition (F_(2,20)_ = 7.89, *P* = 0.011).

### Imaging results

**Figure 4:**
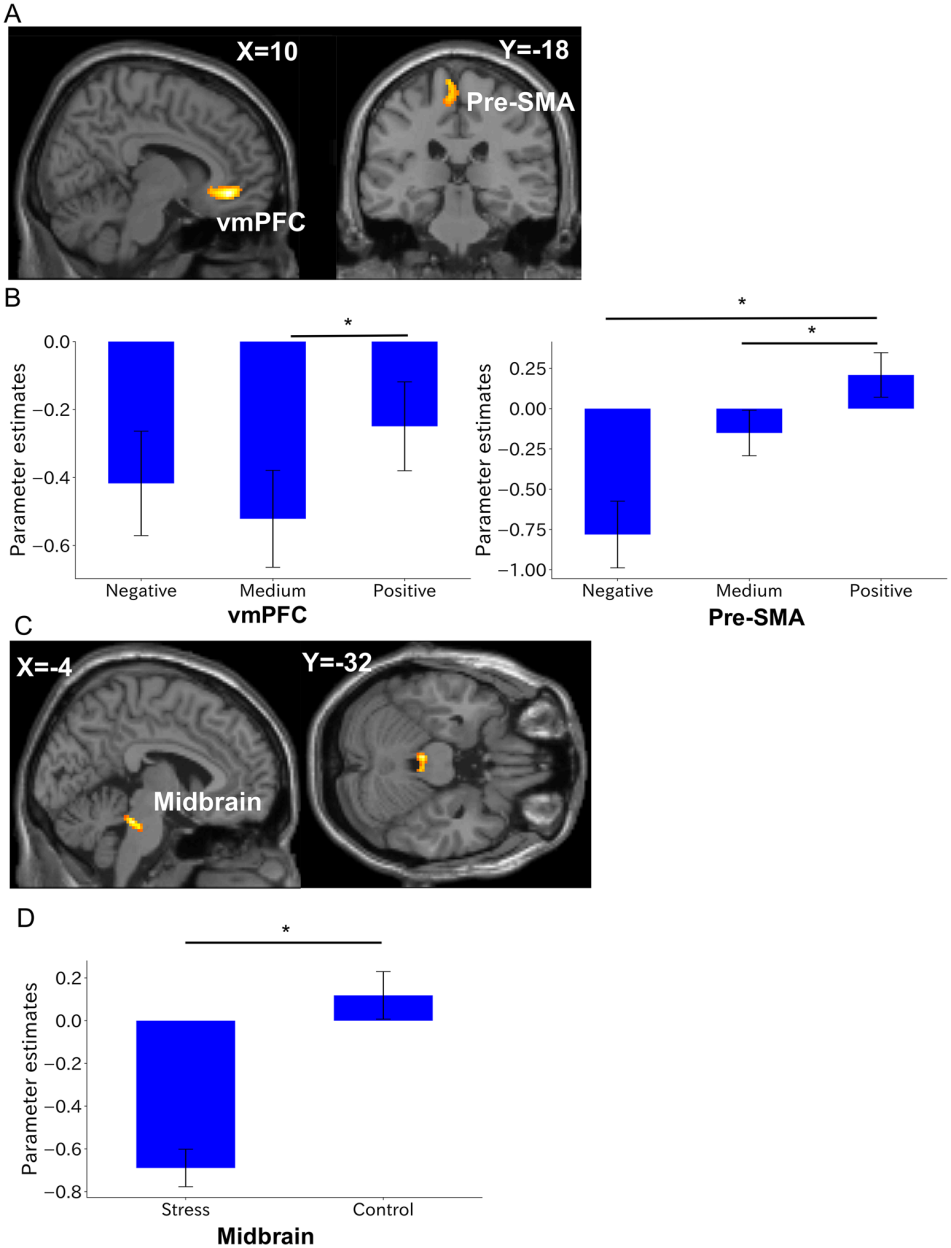
Whole-brain analysis. (**A**) Clusters showing a significant main effect of valence (negative, medium, positive) during the image display period. (**B**) *Post-hoc* analyses of parameter estimates of the image display period sorted by valence in the stress condition for the vmPFC and the pre-SMA. (**C**) Clusters showing a significant main effect of condition (stress, control) during the evaluation period. (**D**) *Post-hoc* analyses of parameter estimates of the evaluation period sorted by valence in the stress condition for the midbrain. These results were obtained with a cluster-defining threshold of *P* < 0.001 and cluster probability of *P* < 0.05 and were FWE-corrected. *Survived Bonferroni correction for the number of elements in the condition. Error bars represent s.e.m. The images were created by using the SPM software (SPM12, https://www.fil.ion.ucl.ac.uk/spm). Clusters were overlayed on the T1-weighted template image (single_subj_T1.nii).

**Figure 5:**
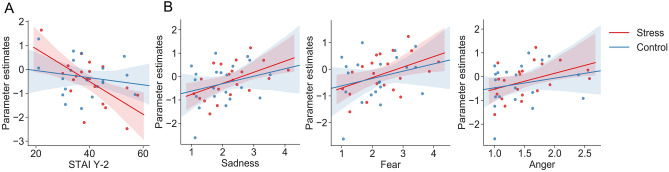
Correlation analysis in the vmPFC and pre-SMA. (**A**) Significant correlation between event-related parameter estimates during the image display period in the vmPFC and the STAI Y-2 (trait anxiety) measured at − 40 min (before the TSST) (r =  − 0.62, *P* = 0.0036). (**B**) Significant correlation plot of event-related parameter estimates during the image display period in the left pre-SMA and the evaluation scores of sadness, fear and anger in the stress condition (r = 0.51, *P* = 0.019; r = 0.45, *P* = 0.039; r = 0.42, *P* = 0.059, respectively). Lines and coloured areas are regression lines and 95% confidence intervals, respectively.

For fMRI analyses, we assessed the interaction between valence and condition using a 3 × 2 ANOVA with valence (negative, medium, positive) and condition (stress, control). The right vmPFC (peak at Montreal Neurological Institute (MNI) x, y, z = 10, 40, − 8; F-value = 17.34; cluster size = 330 mm^3^; Fig. 4A,B) and left pre-supplementary motor area (SMA) (peak at x, y, z =  − 6, − 28, 72; F-value = 10.91; cluster size = 162 mm^3^; Fig. 4A,B) were identified as a main effect of valence when participants looked at a picture for 3 s (all result maps were tested with a cluster-defining threshold of *P* < 0.001 and cluster probability of *P* < 0.05, and were FWE-corrected). We performed correlation analysis between parameter estimates (mean regression coefficients of all trials in each condition) in the vmPFC cluster and STAI Y-2 measured 40 min before the TSST. In the stress condition, parameter estimates of the vmPFC showed a significant negative correlation with STAI Y-2 (r =  − 0.62, *P* = 0.0036; Fig. 5A), unlike the value of the control condition (r =  − 0.19, *P* = 0.42; Fig. 5A).

In addition, parameter estimates (mean regression coefficients of all trials in each condition) in the pre-SMA in the stress condition were significantly correlated with sadness (r = 0.51, *P* = 0.019; Fig. 5B left) and fear (r = 0.45, *P* = 0.039; Fig. 5B middle), although anger was marginally significant in the same condition (r = 0.42, *P* = 0.059; Fig. 5B right). However, values in the control condition were not significant for any evaluation score.

Next, we applied the same ANOVA approach to participant subjective evaluations of an image for 20 s. The left midbrain was identified as a main effect of condition (peaks at x, y, z =  − 4, − 32, − 24; F-value = 25.59; cluster size = 145 mm^3^; Fig. 4C,D). All ANOVA results showed no interaction between valence and condition. *Post-hoc* analysis of the results comprised Bonferroni multiple correction for the number of elements in the condition.

### ROI-to-ROI analysis of functional ROIs

**Figure 6:**
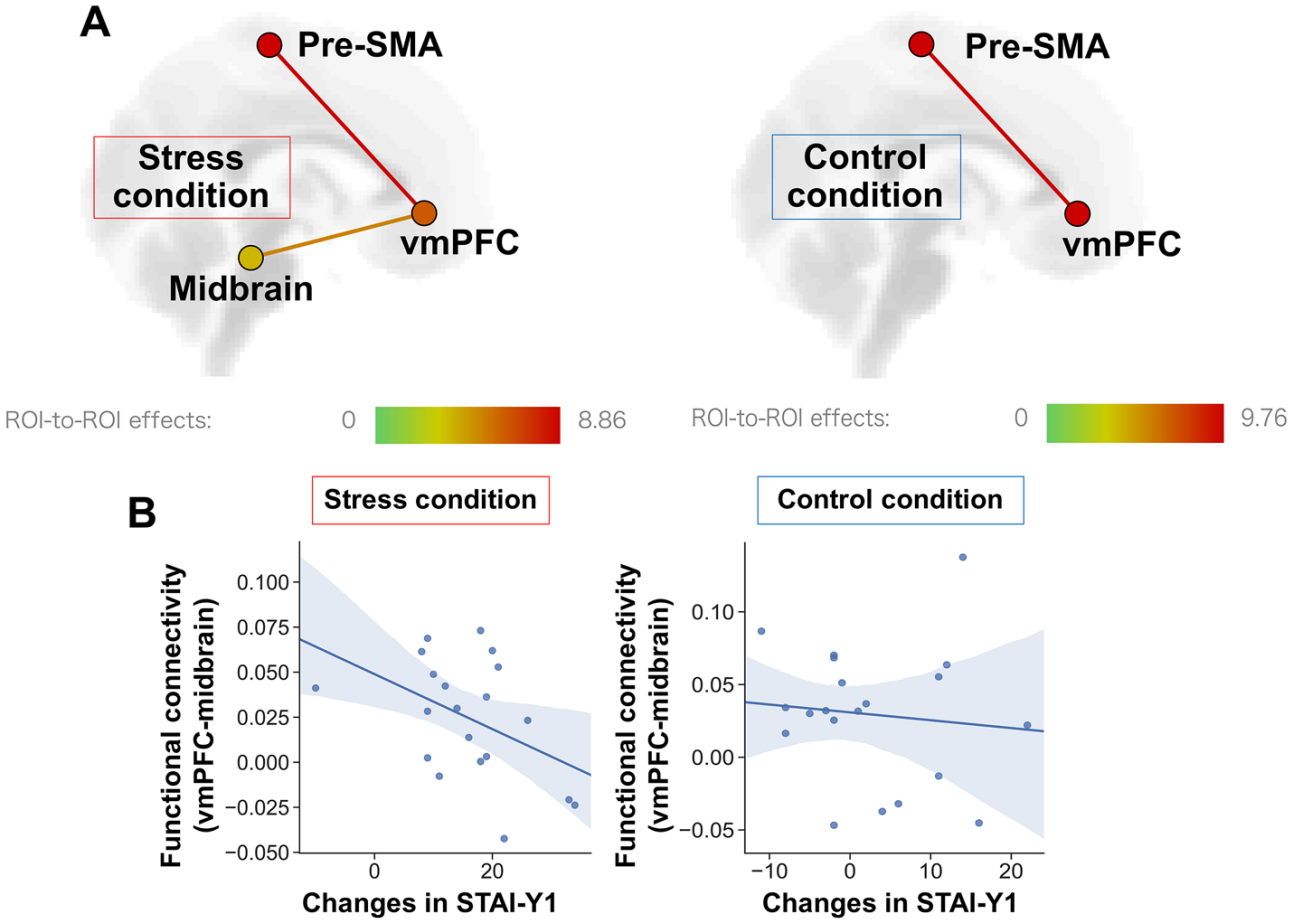
ROI-to-ROI analysis. (**A**) Functional connectivity between targeted areas (left, stress condition; right, control condition). Significant functional connectivity between the vmPFC and midbrain were observed only in the stress condition. The colour bar of ROI-to-ROI effects indicates the F-value. (**B**) Correlation plot of functional connectivity between the vmPFC and midbrain against a change in the state anxiety in the stress condition (r =  − 0.45, *P* = 0.048) and the control condition (r =  − 0.1, *P* = 0.67). Lines and coloured areas are the regression line and the 95% confidence interval, respectively. The images were created by using the CONN software (ver.17.f, https://web.conn-toolbox.org).

To evaluate the relationship between regions involved in emotion evaluation under stress, we conducted functional connectivity analysis of functional ROIs extracted from the imaging results with ANOVA, which were the right vmPFC, left pre-SMA, and left midbrain. ROI-to-ROI correlation analysis was performed to calculate the Z-transformed correlation coefficient to the average BOLD signal in the ROI voxels between time-series signals. We tested the contrast for both stress and control conditions, in which functional connectivity between targets was significantly larger than 0. We found significant functional connectivity between the vmPFC and the midbrain in the stress condition, but not in the control condition (r =  − 0.45, *P* = 0.048; Fig. 6A). We applied correlation analysis using the change in STAI Y-1 (state anxiety) before and after the TSST and the functional connectivity between the vmPFC and midbrain. The functional connectivity in the stress condition showed a significant negative correlation with the change in STAI Y-1, unlike that in the control condition (r =  − 0.1, *P* = 0.67; Fig. 6B).

## Discussion

In an fMRI task after acute social stress, activity of the prefrontal region differed according to image valence category and responded to emotional stimuli, but activity of the midbrain differed according to stress condition and as an emotional response. In addition, activity and connectivity of the vmPFC exhibited individual differences in anxiety in response to stress. Our findings reveal an important mechanism of emotion regulation in response to stress and reflect the level of modulation induced by a stressor.

Consistent with our hypothesis, activity of the vmPFC under stress showed a negative correlation with the STAI-Y2 score (trait anxiety). This individual difference (higher trait anxiety) may reflect a vulnerability to stress. Previous studies showed that stress also potentiates anxiety and impairs emotion regulation^31–33^ and that higher trait anxiety contributes to individual differences in vulnerability to stress^34^. Thus, it seems that this result indicates vulnerability of emotion regulation to stress. However, the vmPFC was detected as a main effect of valence, regardless of stress. Although the vmPFC encodes emotional stimuli or subjective value and regulates negative emotion^9,11,35–37^, emotion regulation of the vmPFC is thought to be related to fear extinction and suppression and to respond to the stimulus itself^22^. Thus, it is not related to the stress per se. Taken together, this suggests that under stress, the vmPFC expresses vulnerability as a result of negative emotion regulation when responding to emotional stimuli.

Here, the pre-SMA showed a main effect of valence. This is consistent with a previous meta-analysis of cognitive emotion regulation showing that the pre-SMA may be involved in execution of regulation^38^. We also observed that the pre-SMA only exhibited a positive correlation with subjective evaluation scores of negative emotions (sadness, fear, and anger) in the stress condition. Previous studies indicated that the pre-SMA reflected the success of emotion regulation for limiting negative context^39^. The pre-SMA was activated in reappraising the emotional evaluation of aversive images, and activation of the pre-SMA predicted the success of reappraisal (i.e., more strongly pre-SMA activated, less negative evaluation). Even though participants in our experiment were not asked to reappraise their emotional rating explicitly, our result suggests the effect of stress on the function of the pre-SMA in cognitive appraisal.

In terms of emotional strength, activity of the midbrain decreased under stress compared with the control condition. The midbrain is involved in not only homeostasis but also emotion^40^.Previous findings that acute stress deactivated limbic regions and that the midbrain was related to the initiation of stress^41^ supports our results. It seems that stress affects our emotional reactivity, deactivates the midbrain, and influences our emotion regulation in homeostasis. Even more interestingly, the MNI coordinates of the peak value of the midbrain, shown as a main effect of stress, nearly corresponded with those of the locus coeruleus (LC) in a recent meta-analysis study^42^. The LC is the main source of noradrenaline and projects to various brain regions^43^. Activity of the LC is independent of affective valence and is associated with acquisition of emotional inputs, storing of emotional memory, and maintenance of the cognitive process^44,45^. In addition, the reduced response of the LC implies resilience promotion, which is consistent with our whole-brain analytical findings for the midbrain^46^. Taken together, one possible interpretation is that the part of the brainstem in which we found a main effect of stress, may collaborate with other brain regions and to help mediate the psychological response to stress. However, we could not identify the peak region accurately, because it is difficult to detect the LC with standard MRI methods^47^.

Finally, in the stress condition, functional connectivity between the vmPFC and midbrain showed individual differences in state anxiety. State anxiety reflects a high stress condition^48^, which supports our results. In addition, cooperation of the vmPFC with limbic regions and the brain stem is important for emotion regulation and stress resilience^18,49^. Indeed, these circuits contribute to negative emotion processing, motivation in behaviour, and fear extinction^50–52^. Recent human MRS studies suggest neurochemical functions of the vmPFC in emotion processing with these circuits. Local GABA levels in the vmPFC exhibited this relation in functional connectivity between vmPFC and the amygdala and individual’s anxiety level^15,16^. However, our findings could not detect effects of valence and stress on limbic regions, especially the amygdala, which is involved in emotion processing. Previous studies indicated that the connection between the vmPFC and amygdala involves negative emotion^12,50^. In addition, brain regions involved in negative emotion and stress responses can overlap^53^. Although the amygdala is thought to be involved in emotion processing, it responds strongly to emotion processing stimulated by facial expressions^54^. The stimulus sets used in our experiment were not specific to facial expressions. They included various categories of stimuli, and stimulus valence scores were balanced. That may be why activity of the amygdala has not been observed (Table 1).

**Table 1:** Numbers of images used in the NAPS BE, by valence and category.

Category/valence	Control condition			Stress condition		
	N	M	P	N	M	P
Animals	3	5	7	4	5	6
Faces	5	5	5	5	5	5
Landscapes	0	3	13	0	3	13
Objects	5	5	5	5	5	5
People	6	4	5	5	4	6
Total	19	22	35	19	22	35

*N* negative-valence image, *M* medium-valence image, *P* positive-valence image.

Our finding that functional connectivity between the vmPFC and midbrain shows individual differences in emotion regulation in response to stress provides new insight into treatments aimed at improving stress vulnerability and suppressing psychiatric disorders, such as major depressive disorder. From the standpoint of emotion regulation, some psychiatric disorders are thought to be characterised by a vulnerability in emotion regulation, and emotion regulation is considered essential for mental health^55,56^. Our findings will help to measure stress resilience for regulation of negative emotions. For psychiatric disorders, a recent animal study of deep brain stimulation (DBS) indicated that under DBS, the vmPFC affects the LC and exerts antidepressant-like effects^57^. In addition, functional connectivity real-time neurofeedback has been applied to some psychiatric disorders^58,59^. Future research is needed to establish new biomarkers for certain psychiatric disorders using neurofeedback and DBS.

The generalizability of our results is limited by the sequence of experimental conditions. Participants first completed the stress condition, which was followed by the control condition. As a result, a framing effect may have occurred in our experiment. However, participants were exposed to acute stress in our study and they completed the control condition at least 2 days after the stress test. In terms of the brain network and neuroendocrine level in response to stress, recovery from acute stressors occurs over several hours^60^. Thus, we believe that prior stress did not affect the control condition. However, we cannot completely exclude the possibility that it may have had a framing effect. Accordingly, future studies that randomly separate stress and control conditions are needed. Another limitation is that we enrolled only male participants in this study. Female stress responses are altered by menstrual cycles and oral contraceptive use^25,26^. Previous studies measuring stress biomarkers have also carefully examined their effects. For example, in a recent systematic review of TSST studies that tested stress biomarkers in saliva^26^, 12 of 35 studies reviewed included only males. However, previous studies showed that the PFC response to stressors differs between male and female rats^61^. The PFC is involved in emotion regulation, but its activity during emotion regulation differs between men and women^62^. Taken together, sex differences may cause a specific emotion regulation effect in response to stress. Thus, the generalizability of our results to females remains an open question.

## Conclusion

To summarise, under acute social stress conditions, prefrontal regions, for example, the vmPFC and pre-SMA, showed individual differences in emotion regulation, such as subjective evaluation and trait anxiety. In addition, functional connectivity between the vmPFC and the midbrain LC showed a negative correlation with variation in the acute social stress effect. Our results suggest that the functions of the network including the vmPFC in emotion regulation is affected by stress depending on the individuals' level of reaction to the stress.

## Methods

### Participants

Twenty-one right-handed healthy adult males (mean age ± SD, 23.10 ± 1.81 years; range, 21–28 years) participated in our experiments. Since female stress responses are altered by menstrual cycles^25,63^, only males were recruited. People without psychiatric disorders, claustrophobia, or neuroendocrine disorders were recruited. Participants were asked if they were taking medication (2 weeks before experiments). They were requested not to consume caffeine or citrus juice for at least 4 h before experiments. They also refrained from heavy exercise, eating/drinking and smoking for at least 2 h before experiments, and from alcohol for 24 h before experiments. Due to an omission in the State-Trait Anxiety Inventory-JYZ (STAI-JYZ), one participant was excluded from correlation and statistical analyses involving the STAI-JYZ. All participants provided informed consent prior to the experiment. All experimental procedures were approved by ATR Review Board Ethics Committee. All procedures were performed in accordance with relevant guidelines and regulations.

### Experimental procedure

All participants underwent the stress condition on the first experimental day and the control condition on the second experimental day. The maximum interval between the first and second experimental days was 5 months. On the first experimental day, an experimenter explained the ethical and safety aspects of the study to all participants. After informed consent was obtained, participants rested for 20 min for both stress and control conditions. They then underwent the stress induction test for 13 min (see ‘Stress induction test’ for details). After the stress induction test, participants entered the fMRI scanner and performed the emotional task (comprising 76 trials separated into four blocks). We repeatedly measured the STAI-JYZ and salivary alpha-amylase before, during and after the stress induction test and fMRI task (see Fig. 1A for the exact timing of measurements). In keeping with previous work^64^, salivary alpha-amylase was measured between 14:10 and 16:40 to minimise diurnal variation of alpha-amylase secretion. Thus, we conducted all experiments between 13:30 and 17:30.

### Stress induction test

All participants underwent the TSST according to a previously published protocol^27,28^. On the first day, participants prepared for a job interview (5 min), completed the job interview speech (5 min), and performed a difficult arithmetic task (3 min) (stress condition). On the second day, participants completed a placebo version of the TSST comprising free speech (5 min), speaking (5 min) and an easy arithmetic task (3 min) (control condition). Both tests were conducted in a small room that was separated from the fMRI scanning room. During the stress condition, participants were interviewed by clinical psychologists (certified in Japan).

### Emotion evaluation in the fMRI task

The fMRI task started 33 min after the start of the TSST. In the MRI scanner, participants looked at natural photographic images and evaluated the strengths of their emotions and preferences for the images (Fig. 1B). After fixation was displayed (1 s), an image was displayed (3 s), and participants indicated their emotional response to the image by moving the cursor on a 7-point Likert scale (pressing the left button moved the cursor left; 1 = not at all, 7 = very much). Participants indicated one of six emotions (happiness, sadness, fear, anger, disgust, surprise) and their preference for the image during a 20-s period. Each trial lasted 24 s. The experiment comprised four sessions (about 30 min), and 19 images were displayed in each session.

Stimulus images comprised 76 images of different valences selected from the Nencki Affective Picture System Basic Emotion (NAPS BE)^65,66^. Valence scores of NAPS datasets are set from 1 to 9. Images were selected from three valence categories by reference to a previous study^65^ of the NAPS (1–4 points, negative; 4–6 points, medium; 6–9 points, positive). In addition, to prevent the serial position effect, which is the tendency of participants to learn when continuously shown the same category and image valences, the number of continuous presentations was limited to two for the same valence and three for the same category. Numbers of images for each valence and category are detailed in Table 1. In both the stress and control conditions, the experimental procedure was the same, and image stimuli used in the task are shown in Table 1.

The analytical design comprised within-participant factors of two conditions (stress and control conditions) and three image valences (negative, medium and positive), and the fMRI and evaluation analyses used the factors in a full factorial design and two-way repeated measures ANOVA. An overview of each trial is shown in Fig. 1B (see ‘fMRI procedure’ in “Methods” for fMRI analysis information).

### Measurement and analysis of salivary alpha-amylase

To evaluate the effect of stress on physiological responses, we measured alpha-amylase in saliva with a salivary amylase monitor (NIPRO, Japan). Saliva can easily be collected with this device and it only requires 1 min to determine alpha-amylase levels. With onset (± 0 min) set as the start of the TSST, saliva was measured in all participants at − 10 min, + 5 min, + 10 min, + 20 min and + 30 min. A paired *t*-test was used to calculate the percent change between − 10 min and + 10 min.

### Measurement and analysis of the STAI

To evaluate effects of stress on psychological responses, we used the STAI, which comprises a 20-item scale for measuring state and trait levels of anxiety^67^. Participants selected a score from 1 to 4 (where 1 = not at all, 4 = very much). We added scores for each item. We used the STAI-JYZ, an official Japanese translated version of the STAI (form-Y). STAI Y-1 (state level) is used to examine acute responses to anxiety, whereas STAI Y-2 (trait level) can measure a stable tendency of the response to a fear stimulus^29^. Measurement times were before the TSST (− 40 min), after the TSST (+ 13 min), and after the fMRI task (+ 80 min). We applied two-way repeated measures ANOVA to test for effects of time on the measure and on stress.

### MRI data acquisition

A 3-T MAGNETOM Prisma MRI system (Siemens) was used to acquire both structural T1-weighted images (repetition time = 2250 ms, echo time = 3.06 ms, flip angle = 9°, thickness = 1 mm, field of view = 256 mm, slice gap = 0 mm) and T2*-weighted echo planar images (repetition time = 2000 ms, echo time = 30 ms, flip angle = 80°, slices = 56, thickness = 2.5 mm, field of view = 200 mm, slice gap = 0 mm, multi-slice mode, interleaved).

### MRI data pre-processing and statistical analysis

All MRI data were converted from DICOM format to NIfTI format. During the process, for the T2* image data of the fMRI task (and not for the T1-weighted image data), the first five scans were excluded as dummy scans. Data were pre-processed using Statistical Parametric Mapping 12 (SPM12, update revision number 7487, Wellcome Department of Cognitive Neurology, London, UK, https://www.fil.ion.ucl.ac.uk/spm) toolbox and MATLAB 2018a (version 9.4.0, The MathWorks, Inc., Natick, Massachusetts, https://matlab.mathworks.com). Images were realigned to the intermediate image as a reference, and volumes underwent segmentation and normalisation to MNI space and were spatially smoothed with an 8-mm full-width at half-maximum Gaussian kernel.

A general linear model (GLM) was applied to pre-processed data. We focused on BOLD signals during two periods: the 3-s image display and the 20-s evaluation. We conducted first-level GLM analyses with a separate designed matrix for those two periods. In each period, all trials were sorted based on image valence (negative, medium and positive) and were defined by three box-car regressors with a duration of 3 s (image display periods) or 20 s (evaluation periods). These factors were convolved with a hemodynamic response function (HRF). Realignment parameters (three translations and three rotations) were added as multiple regressors of head movement. In total, we defined four first-level design matrices for each participant: two periods (image display, evaluation) × two conditions (stress, control). For second-level analysis, we used a flexible factorial model with condition (stress, control) and valence (negative, medium, positive). The model was separately applied to each period of the image display (3 s) and evaluation (20 s). All results of the second-level analysis were corrected using cluster-level inference (a cluster-defining threshold of *P* < 0.001, cluster probability of *P* < 0.05, FWE-corrected).

### Functional connectivity analysis

Functional connectivity analysis was performed using CONN (ver.17.f, https://web.conn-toolbox.org). From results of the flexible factorial model in fMRI analysis, three brain regions were used as functional ROIs, which comprised the vmPFC, pre-SMA and midbrain. ROIs of the pre-SMA and vmPFC were defined by clusters detected from the flexible factorial model, but the midbrain was extracted from a 5-mm radius area around the peak coordinates of the midbrain (MNI: x, y, z =  − 4, − 32, − 24). First, pre-processing and denoising were applied. Pre-processing comprised realignment and unwarp, slice timing correction, segmentation, normalisation, outlier detection, and smoothing (smoothing kernel FWHM of 8 mm). Outlier detection was applied to identify scans with motion larger than the 95% percentile, and these outliers were scrubbed in denoising. Denoising was performed by two methods: 1) linear regression of potential confounding factors in the BOLD signal, including realignment parameters and scrubbing extracted from outlier detection, and 2) temporal band-pass filtering. Next, first-level analysis was performed using regressors of valence and stress conditions in each trial (analytical matrix settings were the same as those of the first-level analysis of SPM12). Regressors were convolved with HRF. ROI-to-ROI connectivity analysis was performed to calculate the Z-transformed correlation coefficient for the averaged BOLD signal across all voxels in the ROI. The duration of the analysis was set to that of the evaluation periods (20 s in each trial). We tested whether functional connectivities between target ROIs were significantly larger than 0 under each of the stress and control conditions.

## Data Availability

Participant fMRI data supporting the conclusions of this paper are not publicly available because they contain information that could compromise research participant privacy.
